# Prescribing Antibiotics in Public Primary Care Clinics in Singapore: A Retrospective Cohort Study

**DOI:** 10.3390/antibiotics12040762

**Published:** 2023-04-16

**Authors:** Sky Wei Chee Koh, Vivien Min Er Lee, Si Hui Low, Wei Zhi Tan, José María Valderas, Victor Weng Keong Loh, Meena Sundram, Li Yang Hsu

**Affiliations:** 1 National University Polyclinics, National University Health System, Singapore 609606, Singapore; 2 Division of Family Medicine, Yong Loo Lin School of Medicine, National University of Singapore, Singapore 119228, Singapore; 3 School of Biological Sciences, Nanyang Technological University, Singapore 637551, Singapore; 4 Saw Swee Hock School of Public Health, National University of Singapore, Singapore 117549, Singapore

**Keywords:** antibiotic, general practice, antibiotic usage, antibiotic prescription rates, antibiotic prevalence, primary care, antimicrobial resistance

## Abstract

Background: Antibiotic prescription practices in primary care in Singapore have received little scholarly attention. In this study, we ascertained prescription prevalence and identified care gaps and predisposing factors. Methods: A retrospective study was conducted on adults (>21 years old) at six public primary care clinics in Singapore. Prescriptions >14 days were excluded. Descriptive statistics were used to showcase the prevalence data. We used chi-square and logistic regression analyses to identify the factors affecting care gaps. Results: A total of 141,944 (4.33%) oral and 108,357 (3.31%) topical antibiotics were prescribed for 3,278,562 visits from 2018 to 2021. There was a significant reduction in prescriptions (*p* < 0.01) before and after the pandemic, which was attributed to the 84% reduction in prescriptions for respiratory conditions. In 2020 to 2021, oral antibiotics were most prescribed for skin (37.7%), genitourinary (20.2%), and respiratory conditions (10.8%). Antibiotic use in the “Access” group (WHO AWaRe classification) improved from 85.6% (2018) to 92.1% (2021). Areas of improvement included a lack of documentation of reasons for antibiotic use, as well as inappropriate antibiotic prescription for skin conditions. Conclusion: There was a marked reduction in antibiotic prescriptions associated with the onset of the COVID-19 pandemic. Further studies could address the gaps identified here and evaluate private-sector primary care to inform antibiotic guidelines and the local development of stewardship programs.

## 1. Introduction

Antimicrobial resistance is widely recognized as a global public health threat [1]. No new classes of antibiotics have been discovered in the past 30 years, and prescription rates are at an all-time global high. This threatens our ability to respond effectively to the global and enduring threat of infectious diseases [2].

Singapore launched its National Strategic Action Plan on Antimicrobial Resistance in 2017 in response to the Global Action Plan for antimicrobial resistance developed by the World Health Assembly [2,3]. This led to the setup of antimicrobial stewardship programs across all public restructured hospitals. A similar initiative, however, is lacking in primary care [4]. Despite electronic prescribing being implemented in most healthcare settings in Singapore, prescription data remains difficult to access, monitor, and regulate [5]. While primary care accounts for 80% of antibiotic prescription in developed countries, 50% of these prescriptions are deemed inappropriate [6]. As healthcare in Singapore reforms towards a population health model called Healthier Singapore [7], this provides an excellent opportunity to launch a primary care antimicrobial stewardship program. Studying the existing data in public primary care institutions could shed light on the current practices, and act as a first step in this momentous push toward appropriate antimicrobial usage in the community.

During the COVID-19 pandemic, changes in patients’ behavior in terms of seeking healthcare and physicians’ prescription patterns may have affected community antibiotic prescription rates [8]. Several studies that have been performed in developed countries revealed a general trend of a reduction in antibiotic prescription in primary care during the pandemic [9,10]. A local study performed in an inpatient setting showed a reduction in antimicrobial prescriptions in 2020 compared to before the pandemic [11]. To date, no study has been conducted concerning antibiotic prescriptions in primary care in Singapore post-COVID-19 pandemic; hence, there is a need to replicate the abovementioned study in the outpatient primary care setting.

In our study, we aim to examine the current patterns of antibiotic prescriptions for adults in primary care, as well as to identify potential care gaps for improvement, and factors influencing these gaps. We hope that this will pave the way for the development of local antibiotic guidelines within primary care and improve governance and stewardship in the post COVID-19 era, toward the creation of a Healthier Singapore.

## 2. Method

### 2.1. Data Source and Study Population

A retrospective observational study was conducted using data extracted from electronic health records (CPSS2 and EPIC) of patients from 6 public primary care clinics (National University Polyclinics) in Singapore, from 2018 to 2021. This included April 2020, which was the peak of the COVID-19 pandemic in Singapore [12]. De-identification was performed by a centralized, trusted third party (institution research office) before passing over to study team for analysis. The study included patients above 21 years of age who visited these 6 clinics and were prescribed an oral or topical antibiotic. Patients on long-term antibiotics for prophylaxis or treatment for more than 14 days were excluded.

Variables included patient demographics, visit diagnoses, presence of chronic diseases, such as diabetes mellitus and chronic kidney disease, antibiotic name and class, and prescriber information, such as place of practice, number of years of practice, training location, and family physician’s accreditation status. Visit diagnoses in clinics were coded using the International Classification of Disease (ICD-10). Institution level data on the total number of visits for each visit diagnosis were also collected to determine the antibiotic prescription rate for each condition. For the purposes of this study, each antibiotic prescribed equates to 1 antibiotic prescription, regardless of number of visits. 

### 2.2. Diagnosis Categorization and Antibiotic Classifications

To analyze antibiotic prescription by diagnoses, visits prescribed with oral antibiotics were grouped into categories based on the indicated diagnosis. These categories consisted of respiratory, skin, genitourinary, gastrointestinal, infectious disease, and dental conditions. Prescriptions for miscellaneous or chronic disease diagnoses where indications were unable to ascertain were listed as ‘Undefined’. The diagnosis categorization was conducted independently by two family physicians based on the World Health Organization (WHO) International Classification of Diseases 10th revision (ICD-10) [13], and split into conditions whereby antibiotics were often required versus not often required. Discrepancies in categorizations were de-conflicted afterwards. For antibiotic classification, we adopted the 2021 WHO AWaRe classification [14].

**Figure A1:**
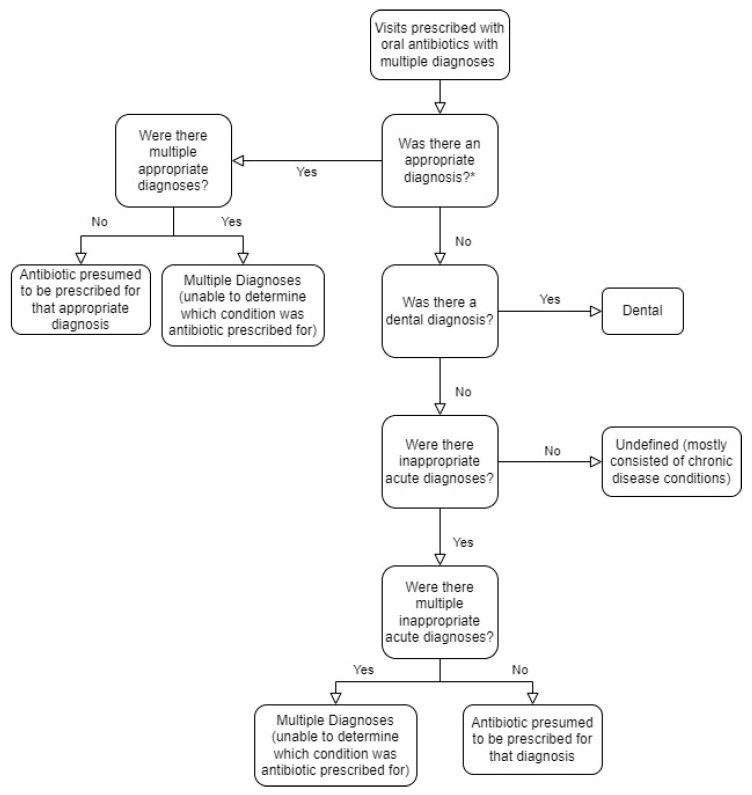
Tiered logic ranking system.

Often, there were oral antibiotics prescribed for visits with multiple diagnoses. We coded a tiered ranking logic system (Figure A1) to select infective conditions over non-infective conditions, and prioritized ranking of conditions in terms of which antibiotics were often required, until each antibiotic prescription belonged to only one category (Table A1). For prescriptions that we were unable to determine the indication of from the listed diagnoses (multiple infective conditions or conditions where antibiotics were often required), they were grouped under ‘multiple diagnoses’.

For visits prescribed with topical antibiotics that were incongruent with the coded diagnosis, we reclassified the diagnosis such that they were prescribed for their indicated conditions and route (i.e., skin topical antibiotics prescribed for skin conditions). In the case of topical ciprofloxacin, which can be used as an eye or ear drop, we differentiated them by the prescribed dosage, duration, and route of application.

Data from 1 clinic were analyzed for the prescription rate of oral antibiotics, but was excluded from other analyses as the clinic was newly built and lacked data before 2021. All antibiotics prescribed by dentists were assumed to be for dental conditions. To ensure data validity and accuracy of ranking classification, 100 case notes were randomly selected and extracted for audits. All information was true and corresponded to the diagnoses and antibiotic characteristics that were extracted. This also ensured and validated the accuracy and robustness of the tiered ranking logic system in diagnosis selection, topical antibiotic diagnosis reclassification, and ciprofloxacin eye and ear drop dichotomization.

### 2.3. Statistical Analysis

Rstudio (R version 4.2.0), IBM SPSS Statistics Version 29.0 and Microsoft Excel 2010 were used in data cleaning and analysis. *p*-value of <0.05 in the two-sided test was considered statistically significant. Descriptive statistics were performed, and numerical variables were represented as mean with standard deviations, or n (%) for categorical variables. Antibiotic prescription rate was derived by dividing the number of prescriptions over the total number of patient visits. Segmented regression analysis was performed to describe antibiotic prescription trends before and after the peak of the pandemic. Chi-square tests were used for categorical variables (i.e., gender, race, and presence of chronic conditions) while logistic regression was performed for continuous variables (i.e., patient’s age and physician’s number of years of practice) to analyze antibiotic prescription for undefined conditions, “Watch” group antibiotic prescriptions, topical antibiotic prescriptions with irrelevant diagnoses, and dual antibiotic prescriptions for skin and soft tissue conditions. Subsequently, combined multivariate logistic regression was performed, considering all variables collected on the gaps identified.

### 2.4. Ethical Considerations

The research was conducted in accordance with the Declaration of Helsinki national and institutional standards and approved by the NHG Domain-Specific Review Board (DSRB) on June 2022 (2022/00319). 

## 3. Results

**Figure 1:**
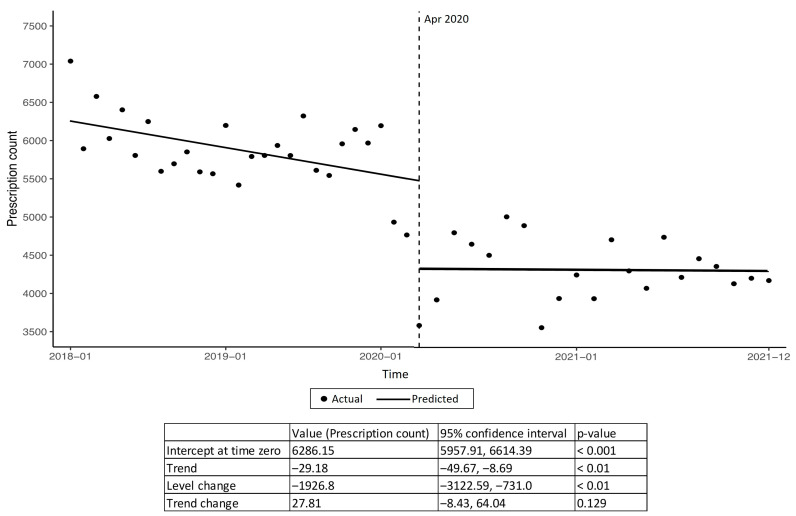
Segmented regression analysis of oral antibiotic prescriptions from 2018 to 2021 (April 2020 was observed as the peak of COVID-19 pandemic in Singapore).

A total of 141,944 oral and 108,357 topical antibiotics were prescribed for 3,278,562 patient visits from 2018 to 2021, giving an overall prescription rate of 4.33% and 3.31%, respectively. For the purposes of analysis, the antibiotic prescriptions from Clinic F were removed due to its introduction in 2021; despite this, Clinic F’s oral antibiotic prescription rate was consistent compared with the other clinics. There was a reduction in the oral antibiotic prescription rate from 5.11% to 3.38% from 2018 to 2021 (Table 1). In particular, we noted a significant reduction in 1926.8 prescriptions (*p* < 0.01) before and after the peak of the COVID-19 pandemic in Singapore in April 2020 (Figure 1). The percentages displayed in the top row of Table 1 were achieved by dividing the total number of antibiotic prescriptions over the total number of patient visits for that year. We noted that this reduction in the antibiotic prescription rate was consistent across all age groups, genders, races, and clinics. The oral antibiotic prescription rates were the highest among younger age groups (22–44) and females. While the majority of antibiotics were prescribed for those of Chinese ethnicity, they had the lowest oral antibiotic prescription rate per clinic visit. The majority of antibiotics were prescribed by family physicians (58.3%) and overseas trained doctors (63.0%).

**Figure 2:**
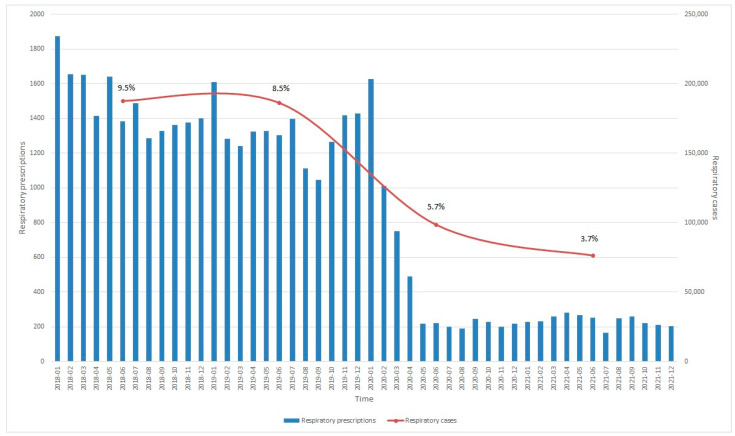
Respiratory visits and antibiotic prescriptions, 2018–2021.

Oral antibiotics were most prescribed for respiratory conditions (29.6%), skin and soft tissue conditions (28.9%), and genitourinary conditions (15.2%) (Table 2). In 2021, skin and soft tissue conditions (37.7%) and genitourinary conditions (20.2%) overtook respiratory conditions to become the top two most common conditions when oral antibiotics were prescribed. This was due to an 84% reduction in respiratory antibiotic prescriptions, with a 5.22% absolute reduction in respiratory condition visits prescribed with oral antibiotics (Figure 2).

Prescriptions for dental, skin and soft tissue, and ear, nose, and throat (ENT) conditions remained stable from 2018 to 2021 (Table 2). While the absolute number of prescriptions for dental conditions remained low, it had the highest percentage of visits that were prescribed with antibiotics (17.8%). The number of visits with multiple infectious conditions reduced from 3.69% in 2018 to 1.67% in 2021. The number of antibiotics prescribed for undefined conditions (diagnoses listed that were non-infectious in nature, such as chronic diseases) rose from 10.8% to 17.2% in terms of the total antibiotics prescribed across 2018 to 2021. While the patient’s age (OR 1.005, 95% CI 1.004–1.006) was associated with antibiotic prescription for undefined conditions, the physician’s years of practice (OR 0.993, 95% CI 0.991–0.995) was found to have an inverse relationship (Table A2). On a multivariate analysis after adjusting for the patient’s age and physician’s years of practice, the female gender (OR 1.12, 95% CI 1.08–1.15), race (*p* < 0.001), presence of diabetes mellitus (OR 1.34, 95% CI 1.29–1.40) and chronic kidney disease (OR 1.31, 95% CI 1.26–1.37), place of practice (*p* < 0.001), and having an accredited family physician (OR 1.16, 95% CI 1.12–1.20) were significantly associated with antibiotic prescriptions for undefined conditions (Table A2).

**Figure 3:**
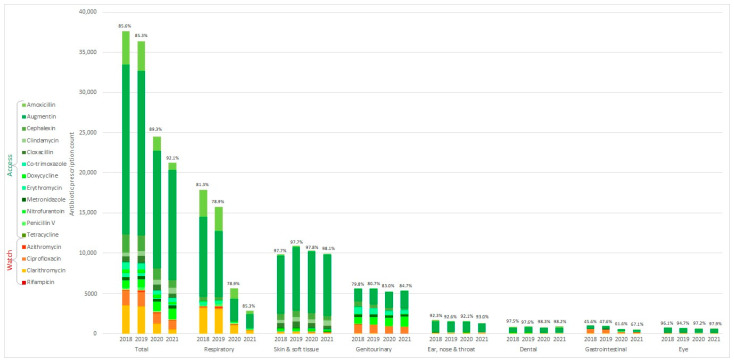
Oral antibiotics classified according to WHO AWaRe, 2018–2021.

Figure 3 describes all the available oral antibiotics split into diagnoses and grouped according to the WHO AWaRE classification. The most common oral antibiotic prescribed from 2018 to 2021 was amoxicillin/clavulanate (58.8%). Skin and soft tissue infections had the highest percentage of antibiotic use in the Access group (98%). The overall increase in the use of antibiotics in the Access group from 85.6% (2018) to 92.1% (2021) was due to the reduction in clarithromycin use, particularly for respiratory conditions. Ciprofloxacin constituted the largest proportion (68%) among the antibiotics used by the Watch group in 2021, of which the majority (70.6%) were prescribed for genitourinary conditions. Ciprofloxacin was 7 and 16 times more likely to be prescribed for genitourinary (OR 7.41, 95% CI 7.05–7.78) and gastrointestinal (OR 16.1, 95% CI 14.9–17.4) conditions, respectively, compared to other conditions. 

The changes in antibiotic prescription habits observed in 2020 and 2021 prompted us to assess the factors contributing to the prescription of Watch group antibiotics. This is showcased in Table A3. On a multivariate analysis (after adjusting for the patient’s age), being male (OR 1.26, 95% CI 1.19–1.35) with gastrointestinal (OR 28.5, 95% CI 24.1–33.8), respiratory (OR 11.9, 95% CI 10.6–13.3), or genitourinary conditions (OR 10.2, 95% CI 9.07–11.4) made one significantly more likely to be prescribed a Watch group antibiotic. Factors such as the physician’s years of experience, being local trained (OR 1.22, 95% CI 1.14–1.30), having an accredited family physician (OR 1.17, 95% CI 1.09–1.25), and the place of practice significantly contributed to the Watch group’s antibiotic prescriptions (Table A3).

Topical antibiotic prescriptions were highest in the younger age groups (age 22–44), with gradual increments of ENT (0.463% to 0.59%) and skin (1.69% to 1.90%) topical antibiotic prescription rates from 2018 to 2021. This is described in Table 3, Table 4 and Table 5, respectively. Topical antibiotic prescriptions also differed between clinics. Topical antibiotics for skin conditions also saw the highest prescriptions among patients with diabetes and chronic kidney disease (Table 5). While the number of topical antibiotic prescriptions differed from clinic to clinic from 2018 to 2021, Clinic C had the highest topical eye and skin antibiotic prescription rate (Table 4 and Table 5). 

Topical antibiotics prescribed without s relevant diagnoses increased most significantly for skin conditions, where the number of prescriptions for non-skin-related diagnoses increased from 5122 (35.2%) in 2018 to 5934 (40.1%) in 2021 (Table A4). Patient factors such as the patient’s age (OR 1.013, 95% CI 1.012–1.015), female gender (OR 1.19, 95% CI 1.15–1.23), Chinese race, presence of diabetes mellitus (OR 1.50, 95% CI 1.44–1.56), and chronic kidney disease (OR 1.23, 95% CI 1.18–1.29) were significantly associated with topical skin antibiotics prescribed for irrelevant diagnoses (Table A5). Factors such as the physician’s years of experience, place of practice, being locally trained (OR 1.07, 95% CI 1.03–1.11), and having an accredited family physician (OR 1.10, 95% CI 1.06–1.14) were significantly associated with inappropriate diagnoses coding during topical antibiotic prescriptions (Table A5).

A significant percentage (35.8%) of same-visit prescriptions of oral and topical antibiotics for skin conditions was also observed compared to oral antibiotic prescriptions. Younger patient ages (OR 0.994, 95% CI 0.993–0.995) and higher years of experience of the physician (OR 1.017, 95% CI 1.015–1.019) were associated with dual antibiotic prescriptions (Table A5). In the multivariate analysis, the female gender (OR 1.13, 95% CI 1.08–1.18), diabetes mellitus (OR 1.06, 95% 1.001–1.11), and the absence of chronic kidney disease (OR 1.11, 95% CI 1.05–1.17) were significant predictors for dual antibiotic prescriptions (Table A6). Factors such as the physician’s years of experience, being overseas trained (OR 1.18, 95% CI 1.13–1.24), having an family physician accredited (OR 1.16, 95% CI 1.11–1.21), and the place of practice were significantly associated with dual antibiotic prescriptions (Table A6).

## 4. Discussion

This observational study is one of the first conducted on oral and topical antibiotic use within primary care clinics in Singapore, showcasing the prevalence of prescription practices and revealing the care gaps. All prescription data were included as they were extracted from a health records database, with no missing data. Diagnoses were mapped to prescription data, with prescription rates calculated based on the overall patient visits, which showcased the actual burden of antibiotic use. Some physician and patient factors affecting antibiotic prescription were also included and analyzed, with meaningful and applicable results. However, this was not an exhaustive list; future studies should focus on this area to help build a more complete picture.

The antibiotic prescription rate reduced with age, which was likely due to a higher proportion of older patients attending for chronic disease visits compared to younger patients. This could also be due to poorer knowledge associated with younger patients in Singapore, leading to more presentations and antibiotic requests [15]. Notably, overall, oral antibiotic prescriptions reduced at a greater proportion compared to visits for respiratory conditions from 2018 to 2021. This was also observed in many countries worldwide [8,10,16,17]. The segmented regression analysis performed showed a significant reduction in antibiotic prescriptions after the peak of the COVID-19 pandemic in April 2020, which was consistent with a previous inpatient local study [11]. This demonstrates that this was due to public health measures, which influenced both outpatient and inpatient antibiotic prescriptions. While previous local studies have suggested possible knowledge gaps among patients and physicians in terms of the variability of prescriptions for respiratory infections [18], the sustained reduction was largely due to increased public awareness and hygiene protocols during the pandemic [19], reduced visits due to altered patient health-seeking behavior, and increased referrals to hospitals for severe disease, which was not presented in primary care [20,21]. In 2020 and 2021, the increased accessibility of testing to the public and usage in primary care clinics for the diagnosis of COVID-19 [22,23], nationwide vaccination drives, and the implementation of vaccine-differentiated safe management measures may have amplified this phenomenon [24]. Future studies should be performed to assess the improvement in knowledge, attitudes, and practices of patients toward antibiotic use pertaining to respiratory infections to compared with the pre-COVID-19 pandemic studies [25].

Data from 2021 also showed that skin and genitourinary conditions accounted for the majority (57.9%) of total oral antibiotic prescriptions, highlighting shifts in antibiotic prescription habits among physicians and patient’s antibiotic requests. The high percentage of antibiotic prescriptions for dental conditions could be attributed to our algorithm for diagnosis classification (Figure A1). Further studies are needed to explore the accuracy of these gaps.

Within the clinics, the lack of prioritization in terms of ensuring the accurate coding of diagnosis for antibiotic prescriptions made the assessment and determination of the indications for antibiotic prescriptions challenging. This was evident in the two gaps that we identified: the increase in oral antibiotics prescribed for chronic condition diagnoses and topical skin antibiotic prescriptions with non-skin diagnoses. Similar gaps were discovered in the USA; the antibiotic prescribed was not listed as a diagnostic code in over 50% of cases [26]. We postulate that the similarity in the identified patient and physician factors could be due to a reduced prioritization of accurate coding with an increased consult complexity and diagnostic uncertainty, perceived demand and expectation from certain patient groups (older and female), and a laxity with regulation and dispensing of antibiotics, which differs from practice to practice and physician seniority [27]. Certainly, further research could be performed in this area to ascertain the accuracy and strength of these associations. The potential collinearity assessed between the factors is a limitation of our study, which we found difficult to adjust for. 

While the increase in the “Access” group’s antibiotic prescriptions was due to reduced clarithromycin use in respiratory conditions, the “Watch” group’s antibiotic utility remained high in genitourinary and gastrointestinal conditions. As we discovered that more experienced, locally trained family physicians were more likely to prescribe “Watch” group antibiotics, this could stem from previous outdated local antimicrobial guidelines, in terms of encouraging ciprofloxacin use for urinary tract infections [28]. This local guideline also recommended ciprofloxacin as a first line for male urinary tract infections, which could possibly explain the factors that we identified (male patients and locally trained physicians). Updated antimicrobial guidelines based on latest antibiograms, whilst important, may not positively influence changes in prescription habits due to significant variability between clinics and physicians [29]. Future interventions such as academic detailing and decision support tools may be effective in monitoring “Watch” group antibiotic prescriptions [30]. 

Our study discovered an increasing use of dual oral and topical antibiotics for skin conditions, despite discouragement from international guidelines and studies due to a possible increased risk of antimicrobial resistance [31]. The factors identified as predisposing to dual antibiotic usage were as follows: demand and expectation from certain groups (younger patients), differences in co-morbidities that shaped their perceived severity (CKD), differing practices, and regulations in prescriptions (resulting in discrepancies in doctor experience, training location, seniority level, and place of practice) [27]. Further research could be performed to ascertain the burden of specific skin conditions and address the potential knowledge gaps. 

A potential limitation in this study is selection bias (due to the use of one public primary care cluster in Singapore), which may not be representative of the whole primary care landscape. Our study found that oral antibiotics were prescribed in 3–5% of all patient visits; this was likely an under-estimate, given that a result of 10% was reported in the USA [32]. Studies conducted abroad were larger in scale and encompassed more data [31]. Compared with the national data, the antibiotic prescription patterns in our study cohort were found to be like the other healthcare clusters [33]. In Singapore, private clinics account for 80% of all primary care services, with 86% of consultations being acute consultations [34]. Most clinics adopted the same prescriber dispenser model, so we expect antibiotic prescriptions to be less regulated, and further studies in private practices to better reflect the antimicrobial gaps of care. Missing diagnoses coding and multiple visit diagnoses might affect the robustness of the tiered ranking system in diagnoses classification, resulting in a misrepresentation of antibiotics prescribed for certain diagnoses (such as dental conditions). An indication of antibiotic prescription might not necessarily equate to visit diagnoses; medical record reviews using machine learning could uncover this difference. The factors affecting the gaps identified should be further brainstormed, extracted, and evaluated to formulate a more accurate representation of antibiotic prescription patterns and habits, and these should be further triangulated in subsequent studies. Due to the lack of guidelines, antibiotic appropriateness could not be determined from this study.

## 5. Conclusions

This study showcases the prevalence of antibiotic prescriptions within public primary care in Singapore and their significant reduction, particularly for respiratory conditions during the COVID-19 pandemic. The gaps identified include inaccurate diagnosis coding for oral and topical antibiotic prescriptions, and dual antibiotic use for skin and soft tissue infections. These are associated with certain patient and physician factors. While the usage of “Watch” group antibiotics has decreased, greater emphasis can be placed on antibiotics prescribed for certain diagnoses. This paves the way for further studies in primary care in Singapore and lays the foundation for updated antimicrobial guidelines and stewardship programs in primary care in Singapore.

## Figures and Tables

**Table 1:** Oral antibiotic prescriptions, 2018–2021.

Variable	2018, *n* = 44,047 (5.11%) ^1^		2019, *n* = 42,631 (4.75%)		2020, *n* = 28,977 (3.99%)		2021, *n* = 26,289 (3.38%)	
	*n* (%)	Prescription Rate %	*n* (%)	Prescription Rate %	*n* (%)	Prescription Rate %	*n* (%)	Prescription Rate %
Age, mean (SD)	52 (17)	-	52 (17)	-	53 (18)	-	53 (18)	-
Age group								
22–44	15,107 (34.3%)	7.02%	14,472 (34.0%)	6.54%	9790 (33.8%)	6.07%	8759 (33.3%)	4.67%
45–54	7586 (17.2%)	5.70%	7072 (16.6%)	5.09%	4711 (16.3%)	4.44%	4209 (16.0%)	3.53%
55–64	9598 (21.8%)	4.98%	9244 (21.7%)	4.64%	6244 (21.6%)	3.73%	5452 (20.7%)	2.97%
65–74	7459 (16.9%)	4.47%	7687 (18.0%)	4.22%	5315 (18.3%)	3.24%	4919 (18.7%)	2.66%
>=75	4297 (9.76%)	4.29%	4156 (9.75%)	3.94%	2917 (10.1%)	3.19%	2950 (11.2%)	2.84%
Gender								
Male	20,283 (46.0%)	5.17%	19,639 (46.1%)	4.78%	13,654 (47.1%)	3.99%	11,794 (44.9%)	3.09%
Female	23,764 (54.0%)	5.71%	22,992 (53.9%)	5.33%	15,323 (52.9%)	4.40%	14,495 (55.1%)	3.65%
Race								
Chinese	28,910 (65.6%)	4.78%	28,080 (65.9%)	4.44%	18,920 (65.3%)	3.72%	17,422 (66.3%)	3.21%
Malay	7306 (16.6%)	5.58%	6840 (16.0%)	5.09%	4686 (16.2%)	4.35%	3995 (15.2%)	3.89%
Indian	4697 (10.7%)	6.10%	4685 (11.0%)	5.98%	3313 (11.4%)	4.91%	2968 (11.3%)	4.15%
Others	3134 (7.12%)	6.46%	3026 (7.10%)	5.97%	2058 (7.10%)	3.77%	1904 (7.24%)	3.87%
Diabetes mellitus, *n* (%)	11,835 (26.9%)	-	11,632 (27.3%)	-	8536 (29.5%)	-	7329 (27.9%)	-
Chronic kidney disease, *n* (%)	10,943 (24.8%)	-	10,461 (24.5%)	-	7538 (26.0%)	-	6537 (24.9%)	-
Primary care clinic, *n* (%)								
Clinic A	9917 (22.5%)	5.70%	8513 (20.0%)	4.82%	5819 (20.1%)	4%	5100 (19.4%)	3.24%
Clinic B	11,379 (25.8%)	4.91%	10,174 (23.9%)	4.42%	6594 (22.8%)	3.57%	5718 (21.8%)	3.10%
Clinic C	9185 (20.9%)	5.93%	8873 (20.8%)	5.45%	5733 (19.8%)	4.43%	5175 (19.7%)	3.81%
Clinic D	7262 (16.5%)	3.82%	7604 (17.8%)	3.93%	5540 (19.1%)	3.55%	5060 (19.3%)	3.05%
Clinic E	6304 (14.3%)	5.69%	7467 (17.5%)	5.60%	5291 (18.3%)	4.74%	4629 (17.6%)	3.96%
Clinic F	0 (0%)	-	0 (0%)	-	0 (0%)	-	607 (2.31%)	3.26%
Prescriber								
Family physician	22,887 (52.0%)	-	25,415 (59.6%)	-	17,227 (59.5%)	-	17,212 (65.5%)	-
Locum	4801 (10.9%)	-	4037 (9.47%)	-	2209 (7.62%)	-	1861 (7.08%)	-
Medical officer	4652 (10.6%)	-	3356 (7.87%)	-	2902 (10.0%)	-	1761 (6.70%)	-
Resident physician	11,707 (26.6%)	-	9823 (23.0%)	-	6639 (22.9%)	-	5455 (20.8%)	-
Training location								
Local	15,597 (35.4%)	-	15,861 (37.2%)	-	10,554 (36.4%)	-	10,452 (39.8%)	-
Overseas	28,450 (64.6%)	-	26,770 (62.8%)	-	18,423 (63.6%)	-	15,837 (60.2%)	-

^1^ Prescription rate per year, %.

**Table 2:** Oral antibiotic prescriptions group by visit diagnoses, 2018–2021.

Conditions	2018		2019		2020		2021		Total	
	*n* (%)	% Visits Prescribed Antibiotics	*n* (%)	% Visits Prescribed Antibiotics	*n* (%)	% Visits Prescribed Antibiotics	*n* (%)	% Visits Prescribed Antibiotics	*n* (%)	% Visits Prescribed Antibiotics
Dental	826 (1.88%)	17.7	891 (2.09%)	17.2	782 (2.70%)	16.9	897 (3.41%)	19.4	3396 (2.39%)	17.8
ENT (ear, nose, and throat)	1710 (3.88%)	7.74	1531 (3.59%)	6.80	1535 (5.30%)	7.51	1322 (5.03%)	8.78	6098 (4.30%)	7.61
Eye	770 (1.75%)	2.21	729 (1.71%)	2.19	604 (2.08%)	2.34	560 (2.13%)	2.31	2663 (1.88%)	2.25
Gastrointestinal	1019 (2.31%)	1.06	952 (2.23%)	0.96	497 (1.72%)	0.77	429 (1.63%)	0.75	2897 (2.04%)	0.911
Genitourinary	5592 (12.7%)	18.5	5577 (13.1%)	19.2	5156 (17.8%)	18.4	5299 (20.2%)	11.4	21,624 (15.2%)	16.2
Infectious diseases	51 (0.116%)	2.43	63 (0.148%)	2.10	73 (0.252%)	1.91	49 (0.186%)	1.96	236 (0.166%)	2.07
Respiratory	17,864 (40.6%)	9.52	15,756 (37.0%)	8.47	5611 (19.4%)	5.70	2848 (10.8%)	3.73	42,079 (29.6%)	7.67
Skin and soft tissue	9856 (22.4%)	11.9	10,903 (25.6%)	12.7	10,343 (35.7%)	13.2	9913 (37.7%)	14.0	41,015 (28.9%)	12.9
Multiple diagnoses	1625 (3.69%)	-	1567 (3.68%)	-	809 (2.79%)	-	439 (1.67%)	-	4440 (3.13%)	-
Undefined	4734 (10.8%)	-	4662 (10.9%)	-	3567 (12.3%)	-	4533 (17.2%)	-	17,496 (12.3%)	-

*n* = number of antibiotic prescriptions.

**Table 3:** Topical ENT antibiotic prescriptions, 2018–2021.

Variable	2018, *n* = 3991 (0.463%) ^1^		2019, *n* = 4042 (0.451%) ^1^		2020, *n* = 4274 (0.588%) ^1^		2021, *n* = 4591 (0.590%) ^1^	
	*n* (%)	Prescription Rate %	*n* (%)	Prescription Rate %	*n* (%)	Prescription Rate %	*n* (%)	Prescription Rate %
Age, mean (SD)	52 (17)	-	51 (17)	-	51 (17)	-	51 (17)	-
*Age group, n (%)*								
22–44	1373 (34.4%)	0.638%	1443 (35.7%)	0.652%	1490 (34.9%)	0.924%	1622 (35.3%)	0.866%
45–54	693 (17.4%)	0.521%	733 (18.1%)	0.528%	744 (17.4%)	0.701%	780 (17.0%)	0.654%
55–64	947 (23.7%)	0.492%	885 (21.9%)	0.444%	1037 (24.3%)	0.619%	1050 (22.9%)	0.572%
65–74	652 (16.3%)	0.391%	710 (17.6%)	0.389%	741 (17.3%)	0.452%	812 (17.7%)	0.440%
>= 75	326 (8.17%)	0.325%	271 (6.71%)	0.257%	262 (6.13%)	0.286%	327 (7.12%)	0.315%
*Gender, n (%)*								
Male	1956 (49.0%)	0.499%	1928 (47.7%)	0.469%	2079 (48.6%)	0.608%	2247 (48.9%)	0.589%
Female	2035 (51.0%)	0.489%	2114 (52.3%)	0.490%	2195 (51.4%)	0.631%	2344 (51.1%)	0.590%
*Race, n (%)*								
Chinese	2612 (65.4%)	0.432%	2704 (66.9%)	0.427%	2844 (66.5%)	0.559%	3123 (68.0%)	0.576%
Malay	612 (15.3%)	0.467%	552 (13.7%)	0.411%	611 (14.3%)	0.567%	608 (13.2%)	0.592%
Indian	484 (12.1%)	0.629%	522 (12.9%)	0.667%	521 (12.2%)	0.772%	539 (11.7%)	0.754%
Others	283 (7.09%)	0.583%	264 (6.53%)	0.521%	298 (6.97%)	0.545%	321 (6.99%)	0.652%
Diabetes mellitus, n (%)	978 (24.5%)	-	887 (21.9%)	-	973 (22.8%)	-	983 (21.4%)	-
Chronic kidney disease, n (%)	877 (22.0%)	-	819 (20.3%)	-	823 (19.3%)	-	849 (18.5%)	-
*Primary care clinic, n (%)*								
Clinic A	806 (20.2%)	0.463%	744 (18.4%)	0.421%	792 (18.5%)	0.544%	870 (19.0%)	0.553%
Clinic B	1048 (26.3%)	0.452%	948 (23.5%)	0.412%	916 (21.4%)	0.496%	953 (20.8%)	0.517%
Clinic C	800 (20.0%)	0.517%	845 (20.9%)	0.519%	919 (21.5%)	0.710%	937 (20.4%)	0.689%
Clinic D	758 (19.0%)	0.398%	746 (18.5%)	0.385%	839 (19.6%)	0.538%	970 (21.1%)	0.584%
Clinic E	579 (14.5%)	0.523%	759 (18.8%)	0.569%	808 (18.9%)	0.724%	761 (16.6%)	0.651%
Clinic F	0 (0%)	-	0 (0%)	-	0 (0%)	-	100 (2.18%)	0.538%
*Prescriber, n (%)*								
Family physician	1950 (48.9%)	-	2428 (60.1%)	-	2563 (60.0%)	-	3060 (66.7%)	-
Locum	422 (10.6%)	-	352 (8.71%)	-	314 (7.35%)	-	307 (6.69%)	-
Medical officer	562 (14.1%)	-	365 (9.03%)	-	502 (11.8%)	-	346 (7.54%)	-
Resident physician	1057 (26.5%)	-	897 (22.2%)	-	895 (20.9%)	-	878 (19.1%)	-
*Training location, n (%)*								
Local	1447 (36.3%)	-	1574 (38.9%)	-	1608 (37.6%)	-	1912 (41.6%)	-
Overseas	2544 (63.7%)	-	2468 (61.1%)	-	2666 (62.4%)	-	2679 (58.4%)	-
Prescription rate by diagnosis, %	-	18.1%	-	18.0%	-	20.9%	-	30.5%

^1^ Overall prescription rate, %.

**Table 4:** Topical eye antibiotic prescriptions, 2018–2021.

Variable	2018, *n* = 9703 (1.13%) ^1^		2019, *n* = 9386 (1.05%) ^1^		2020, *n* = 7159 (0.985%) ^1^		2021, *n* = 7040 (0.904%) ^1^	
	*n* (%)	Prescription Rate %	*n* (%)	Prescription Rate %	*n* (%)	Prescription Rate %	*n* (%)	Prescription Rate %
Age, mean (SD)	49 (17)		49 (17)		51 (17)		50 (17)	
*Age group, n (%)*								
22–44	3807 (39.2%)	1.77%	3754 (40.0%)	1.70%	2607 (36.4%)	1.62%	2666 (37.9%)	1.42%
45–54	1762 (18.2%)	1.32%	1548 (16.5%)	1.12%	1240 (17.3%)	1.17%	1225 (17.4%)	1.03%
55–64	2145 (22.1%)	1.11%	2059 (21.9%)	1.03%	1676 (23.4%)	1.00%	1512 (21.5%)	0.823%
65–74	1427 (14.7%)	0.855%	1487 (15.8%)	0.816%	1245 (17.4%)	0.759%	1223 (17.4%)	0.663%
>= 75	562 (5.79%)	0.561%	538 (5.73%)	0.510%	391 (5.46%)	0.427%	414 (5.88%)	0.398%
*Gender, n (%)*								
Male	4656 (48.0%)	1.19%	4633 (49.4%)	1.13%	3605 (50.4%)	1.05%	3567 (50.7%)	0.934%
Female	5047 (52.0%)	1.21%	4753 (50.6%)	1.10%	3554 (49.6%)	1.02%	3473 (49.3%)	0.875%
*Race, n (%)*								
Chinese	6699 (69.0%)	1.11%	6471 (68.9%)	1.02%	4913 (68.6%)	0.965%	4883 (69.4%)	0.901%
Malay	1636 (16.9%)	1.25%	1564 (16.7%)	1.16%	1181 (16.5%)	1.10%	1083 (15.4%)	1.05%
Indian	812 (8.37%)	1.06%	802 (8.55%)	1.02%	627 (8.76%)	0.929%	625 (8.88%)	0.874%
Others	556 (5.73%)	1.15%	549 (5.85%)	1.08%	438 (6.12%)	0.802%	449 (6.38%)	0.912%
Diabetes mellitus, n (%)	1913 (19.7%)	-	1774 (18.9%)	-	1469 (20.5%)	-	1324 (18.8%)	-
Chronic kidney dsease, n (%)	1679 (17.3%)	-	1579 (16.8%)	-	1272 (17.8%)	-	1127 (16.0%)	-
*Primary care clinic, n (%)*								
Clinic A	1956 (20.2%)	1.12%	1792 (19.1%)	1.01%	1478 (20.6%)	1.02%	1396 (19.8%)	0.888%
Clinic B	2456 (25.3%)	1.06%	2155 (23.0%)	0.937%	1358 (19.0%)	0.736%	1286 (18.3%)	0.698%
Clinic C	2024 (20.9%)	1.31%	2036 (21.7%)	1.25%	1672 (23.4%)	1.29%	1538 (21.8%)	1.13%
Clinic D	1918 (19.8%)	1.01%	1818 (19.4%)	0.939%	1376 (19.2%)	0.882%	1529 (21.7%)	0.921%
Clinic E	1349 (13.9%)	1.22%	1585 (16.9%)	1.19%	1275 (17.8%)	1.14%	1156 (16.4%)	0.989%
Clinic F	0 (0%)		0 (0%)		0 (0%)		135 (1.92%)	0.726%
*Prescriber, n (%)*								
Family physician	4862 (50.1%)	-	5803 (61.8%)	-	4377 (61.1%)	-	4778 (67.9%)	-
Locum	1162 (12.0%)	-	853 (9.09%)	-	567 (7.92%)	-	522 (7.42%)	-
Medical officer	1122 (11.6%)	-	725 (7.72%)	-	685 (9.57%)	-	419 (5.95%)	-
Resident physician	2557 (26.4%)	-	2005 (21.4%)	-	1530 (21.4%)	-	1321 (18.8%)	-
*Training location, n (%)*								
Local	3318 (34.1%)	-	3603 (38.4%)	-	2623 (36.6%)	-	2928 (41.6%)	-
Overseas	6385 (65.8%)	-	5783 (61.6%)	-	4536 (63.4%)	-	4112 (58.4%)	-
Prescription rate by diagnosis, %	-	27.9%	-	28.2%	-	27.7%	-	29.1%

^1^ Overall prescription rate, %.

**Table 5:** Topical skin antibiotic prescriptions, 2018–2021.

Variable	2018, *n* = 14,558 (1.69%) ^1^		2019, *n* = 14,445 (1.61%) ^1^		2020, *n* = 14,359 (1.98%) ^1^		2021, *n* = 14,809 (1.90%) ^1^	
	*n* (%)	Prescription Rate %	*n* (%)	Prescription Rate %	*n* (%)	Prescription Rate %	*n* (%)	Prescription Rate %
Age, mean (SD)	56 (18)	-	56 (18)	-	57 (17)	-	57 (17)	-
*Age group, n (%)*								
22–44	3909 (26.9%)	1.82%	3793 (26.3%)	1.71%	3450 (24.0%)	2.14%	3667 (24.8%)	1.96%
45–54	2155 (14.8%)	1.62%	2048 (14.2%)	1.48%	2053 (14.3%)	1.93%	2135 (14.4%)	1.79%
55–64	3292 (22.6%)	1.71%	3231 (22.4%)	1.62%	3283 (22.9%)	1.96%	3273 (22.1%)	1.78%
65–74	3189 (21.9%)	1.91%	3301 (22.9%)	1.81%	3552 (24.7%)	2.17%	3721 (25.1%)	2.02%
>= 75	2013 (13.8%)	2.01%	2072 (14.3%)	1.96%	2021 (14.1%)	2.21%	2013 (13.6%)	1.94%
*Gender, n (%)*								
Male	7556 (51.9%)	1.93%	7414 (51.3%)	1.80%	7547 (52.6%)	2.21%	7550 (51.0%)	1.98%
Female	7002 (48.1%)	1.68%	7031 (48.7%)	1.63%	6812 (47.4%)	1.96%	7259 (49.0%)	1.83%
*Race, n (%)*								
Chinese	10,485 (72.0%)	1.73%	10,557 (73.1%)	1.67%	10,622 (74.0%)	2.09%	11,003 (74.3%)	2.03%
Malay	1826 (12.5%)	1.39%	1726 (11.9%)	1.28%	1691 (11.8%)	1.57%	1629 (11.0%)	1.59%
Indian	1461 (10.0%)	1.90%	1383 (9.57%)	1.77%	1325 (9.23%)	1.96%	1410 (9.52%)	1.97%
Others	786 (5.40%)	1.62%	779 (5.39%)	1.54%	721 (5.02%)	1.32%	767 (5.18%)	1.56%
Diabetes mellitus, n (%)	5166 (35.5%)	-	5174 (35.8%)	-	5450 (38.0%)	-	5230 (35.3%)	-
Chronic kidney disease, n (%)	4574 (31.4%)	-	4509 (31.2%)	-	4598 (32.0%)	-	4356 (29.4%)	-
*Primary care clinic, n (%)*								
Clinic A	2825 (19.4%)	1.62%	2740 (19.0%)	1.55%	2976 (20.7%)	2.05%	2983 (20.1%)	1.90%
Clinic B	3596 (24.7%)	1.55%	3406 (23.6%)	1.48%	3142 (21.9%)	1.70%	3222 (21.8%)	1.75%
Clinic C	3426 (23.5%)	2.21%	3301 (22.9%)	2.03%	2932 (20.4%)	2.27%	2965 (20.0%)	2.18%
Clinic D	3003 (20.6%)	1.58%	3145 (21.8%)	1.62%	3426 (23.9%)	2.20%	3361 (22.7%)	2.03%
Clinic E	1708 (11.7%)	1.54%	1853 (12.8%)	1.39%	1883 (13.1%)	1.69%	1965 (13.3%)	1.68%
Clinic F	0 (0%)	-	0 (0%)	-	0 (0%)	-	313 (2.11%)	1.68%
*Prescriber, n (%)*								
Family physician	7679 (52.7%)	-	8692 (60.2%)	-	8781 (61.2%)	-	10,436 (70.5%)	-
Locum	1641 (11.3%)	-	1198 (8.29%)	-	1039 (7.24%)	-	994 (6.71%)	-
Medical officer	1573 (10.8%)	-	1200 (8.31%)	-	1267 (8.82%)	-	992 (6.70%)	-
Resident physician	3665 (25.2%)	-	3355 (23.2%)	-	3272 (22.8%)	-	2387 (16.1%)	-
*Training location, n (%)*								
Local	5251 (36.1%)	-	5705 (39.5%)	-	5521 (38.5%)	-	6264 (42.3%)	-
Overseas	9307 (63.9%)	-	8740 (60.5%)	-	8838 (61.6%)	-	8545 (57.7%)	-
Prescription rate by diagnosis, %	-	17.6%	-	16.8%	-	18.3%	-	20.9%

^1^ Overall prescription rate, %.

## Data Availability

The data presented in the study are available on request from the corresponding author.

## Appendix A

**Table A1:** List of diagnosis codes and categorizations.

**Respiratory Conditions (Presumed To Be Infective)**							
respiratory tuberculosis unspecified, without mention of bacteriological or histological confirmation	tuberculosis	pneumonia	pneumonia, unspecified	COPD	chronic obstructive pulmonary disease, unspecified	chronic obstructive pulmonary disease (COPD)	CAP (community acquired pneumonia)
asthma-copd overlap syndrome	bronchiectasis	whooping cough, unspecified					
**Respiratory Conditions (Presumed To Be Non-Infective)**							
acute bronchitis, unspecified	acute upper respiratory infection, unspecified	asthma	asthma, unspecified	influenza with other respiratory manifestations, influenza virus identified	upper respiratory tract infection	URTI (acute upper respiratory infection)	COVID-2019: suspect case
URTI	influenza-like illness	acute bronchitis	disorder of respiratory system	respiratory disorder, unspecified	other respiratory conditions	asthma (bronchial)	coronavirus infection, unspecified site
pulmonary embolism without mention of acute cor pulmonale	pulmonary embolism	coronavirus infection	respiration disorder	cough			
**Skin Conditions (Presumed To Be Infective)**							
acne, unspecified	abscess	carbuncle of skin and/or subcutaneous tissue	cellulitis	cellulitis, unspecified	burn of unspecified body region, unspecified thickness	disorder of nail	unspecified diabetes mellitus with foot ulcer due to multiple causes
furuncle of skin or subcutaneous tissue	nail disorder, unspecified	DM foot	open wound of unspecified body region	ulcer of lower limb, not elsewhere classified	burns	skin infection	open wound
diabetic foot ulcer	nail disease	chronic ulcer of lower extremity	acne	FB skin	multiple wounds	cutaneous abscess, furuncle and carbuncle, unspecified	decubitus ulcer and pressure area, unspecified
injury	injury, unspecified	superficial foreign body (splinter) of unspecified body region	other injuries	other breast conditions	burn	wound cellulitis	pressure ulcer
superficial burn	boil	furuncle	paronychia of left thumb	laceration	dog bite	cat bite	skin abscess
folliculitis	foreign body (FB) in soft tissue	mastitis	paronychia of great toe of left foot	paronychia of finger	acute mastitis	paronychia of third toe of right foot	cellulitis of foot, right
breast abscess	erysipelas	foot ulcer due to secondary dm	surgical wound breakdown	superficial foreign body			
**Skin Conditions (Presumed To Be Non-Infective)**							
skin disorder	dermatomycosis	disorder of skin and subcutaneous tissue	disorder of skin and subcutaneous tissue, unspecified	flexural atopic dermatitis	fungal infection	nonscarring hair loss, unspecified	other atopic dermatitis
other psoriasis	scabies	superficial mycosis, unspecified	unspecified contact dermatitis, unspecified cause	viral warts	contusion	warts	eczema
abrasion	pruritus	psoriasis	corn/callus	urticaria	other skin conditions	alopecia	neonatal jaundice
corns and callosities	other specified soft tissue disorders, site unspecified	soft tissue disorder	varicose veins of lower extremities without ulcer or inflammation	varicose veins, legs	dermatitis	atopic dermatitis	neonatal jaundice, unspecified
contact dermatitis	skin abnormalities	sebaceous cyst	varicose veins of lower extremity	viral wart	tinea pedis	corn	asteatotic eczema
lipoma	callus	skin tag	abrasion of heel	callus of hand	squamous cell carcinoma of skin	rash	melanocytic naevi
tinea corporis	ingrowing toenail	ingrowing left great toenail	ingrown left big toenail	IGTN (ingrowing toe nail)	ingrowing right great toenail	ingrown nail of great toe	disorder of skin
lump in neck	granuloma of skin	neck mass	follow-up examination after surgery for other conditions	atherosclerotic pvd with ulceration	tinea unguium	ganglion cyst	ganglion, site unspecified
swelling of left side of face							
**Genitourinary Conditions (Presumed To Be Infective)**							
Gonococcal infection of lower genitourinary tract without periurethral or accessory gland abscess	urinary tract infection	urinary tract infection, site not specified	UTI	unspecified sexually transmitted disease	vaginal discharge	balanitis	sexually transmitted disease
male genital lesion	UTI (urinary tract infection)	BV (bacterial vaginosis)	cystitis	bacterial vaginosis	chronic prostatitis	other venereal disease	
**Genitourinary Conditions (Presumed To Be Non-Infective)**							
Candidiasis	Candidiasis, unspecified	haematuria	unspecified haematuria	disorder of kidney and ureter, unspecified	unspecified condition associated with female genital organs and menstrual cycle	urinary incontinence	unspecified urinary incontinence
urinary calculus, unspecified	sexual dysfunction	other male genital disorders	other gynaecological conditions	dysmenorrhoea	menorrhagia	calculus, urinary tract	other urinary disorders
abnormal uterine and vaginal bleeding, unspecified	calculus, urinary	disorder of kidney and ureter	genital herpes (recurrent)	undescended testicle, unspecified laterality, unspecified site	unspecified sexual dysfunction, not caused by organic disorder or disease	disorder of menstrual bleeding	bph associated with nocturia
BPH (benign prostatic hyperplasia)	phimosis of penis	congenital anomaly of urinary system	congenital anomaly of female genital system	uterine fibroid	PCOS (polycystic ovarian syndrome)	calculus of ureter	Candidiasis of vagina
Candidiasis of vulva and vagina	vulvovaginal Candidiasis	prolapse of female pelvic organs	menopause	urinary disorder	urine abnormality	benign essential microscopic haematuria	urolithiasis
albuminuria	bladder disorder	fibroid	post-menopausal atrophic vaginitis	atrophic vaginitis	disorder of male genital organ	disorder of male genital organs, unspecified	disorder of female genital organs
female genital disorder	renal stone	AKI (acute kidney injury)	congenital malformation of urinary system, unspecified				
**Gastrointestinal Conditions (Presumed To Be Infective)**							
anorectal abscess	other gastroenteritis and colitis of unspecified origin	peptic ulcer, unspecified as acute or chronic, without haemorrhage or perforation	peptic ulcer disease	acute appendicitis unspecified	perineal abscess	perianal abscess	
**Gastrointestinal Conditions (Presumed To Be Non-Infective)**							
GORD (gastro oesophageal reflux disease)	gastroesophageal reflux disease	gastroenteritis, acute	anal fissure, unspecified	anal fistula	dysphagia	functional dyspepsia	gastroduodenitis, unspecified
gastro-oesophageal reflux disease without oesophagitis	haemorrhoids	irritable bowel syndrome without diarrhoea	noninfectious gastroenteritis and colitis	unspecified abdominal hernia without obstruction or gangrene	unspecified haemorrhoids without complication	incontinence/enuresis	constipation
gerd	piles	gastritis	other git conditions	dyspepsia	disease of intestine, unspecified	foreign body in alimentary tract, part unspecified	other and unspecified abdominal pain
abdominal pain	dyspepsia and disorder of function of stomach	IBS	vomiting of pregnancy, unspecified	disorder of intestine	anal fissure	gastroduodenitis	abdominal hernia
irritable bowel syndrome	gastroenteritis	piles (haemorrhoids)	diarrhoea	intestinal disorder	GERD (gastroesophageal reflux disease)	foreign body in alimentary tract	perianal fistula
intestinal bleeding	inguinal hernia	enteritis,ge	enteritis				
**Infectious Disease Conditions (Presumed To Be Infective)**							
infectious disease	other and unspecified infectious diseases	other infections (non-notifiable)					
**Infectious Disease Conditions (Presumed To Be Non-Infective)**							
dengue fever [classical dengue]	enteroviral vesicular stomatitis with exanthem	varicella without complication	zoster without complication	herpes zoster	dengue	chickenpox	herpes zoster without complication
unspecified arthropod-borne viral fever	varicella uncomplicated	viral illness	fever	hand, foot and mouth disease (HFMD)	parasite infection	viral hepatitis	
**Dental Conditions**							
necrosis of pulp	reversible pulpitis	irreversible pulpitis	periodontitis	dental caries	gingivitis	defective dental restoration	retained dental root
combined periodontal and endodontic lesion	caries	tooth abrasion	arrested dental caries	fracture of crown, enamel, and dentin of tooth without pulp exposure	dentine hypersensitivity	fracture of crown, enamel, and dentin of tooth with pulp exposure	abrasion of teeth
fracture of dental restoration	gingival hyperplasia	fracture of tooth	teeth problem	gum disease	disorder of teeth and supporting structures	disorder of teeth and supporting structures, unspecified	other and unspecified lesions of oral mucosa
teeth & supporting structure disease	disease of salivary gland, unspecified	disorder of oral soft tissue	disorder of salivary gland	oral soft tissue disease	abscess of buccal space of mouth	impacted third molar tooth	cracked tooth
impacted teeth with abnormal position	oral infection	horizontal fracture of tooth	vertical fracture of root of tooth	peri-implantitis, dental	fascial space infection of mouth	infection of buccal space	fracture of root of tooth
periodontal abscess	pericoronitis	symptomatic periapical periodontitis	alveolar osteitis	symptomatic irreversible pulpitis	chronic apical abscess	pulpal necrosis	apical abscess
**ENT Conditions (Presumed To Be Infective)**							
acute tonsillitis	disorder of ear	disorder of ear, unspecified	otitis externa, unspecified	otitis media, unspecified	otitis externa	otitis media	acute sinusitis
chronic sinusitis	disorder of nose	acute infective otitis externa	infective otitis externa	external otitis of left ear	sinus disorder	sinusitis	cervical lymphadenopathy
**ENT Conditions (Presumed To Be Non-Infective)**							
allergic rhinitis, unspecified	epistaxis	allergic rhinitis	chronic mucoid otitis media	chronic secretory otitis media	foreign body in ear	foreign body in nostril	impacted cerumen
ear wax	other ear conditions	FB ear	hearing loss, unspecified	hearing loss	mumps without complication	problems with hearing	foreign body in nose
MUMPS							
**Eye Conditions (Presumed To Be Infective)**							
FB eye	conjunctivitis	chalazion	conjunctivitis, unspecified	eyelid disorder	disorder of eyelid, unspecified	foreign body on external eye, part unspecified	foreign body in external eye
blepharitis of eyelid of left eye	external hordeolum	infected eye lid	hordeolum	blepharitis	stye external	stye	periorbital cellulitis
**Eye Conditions (Presumed To Be Non-Infective)**							
disorder of eye	disorder of eye and adnexa, unspecified	disorder of refraction, unspecified	glaucoma, unspecified	refractive vision	cataracts	other eye conditions	cataract, unspecified
disorder of eyelid	congenital anomaly of eye	eye disorder	cataract	dry eyes	glaucoma	eye discomfort	disorder of refraction and accommodation
blindness of one eye	conjunctival haemorrhage	H/O subconjunctival haemorrhage	vitreous haemorrhage of left eye	congenital malformation of eye, unspecified			
**Unspecified**							
Impaired glucose regulation	Bursitis	Encounter for education	Acquired absence of leg at or below knee	Generalised osteoarthritis	Routine child health examination	Osteoporosis-fracture-vertebral	Chronic renal failure
Impaired glucose regulation without complication	Acquired absence of foot	Status post below-knee amputation	Administrative encounter	Gynaecological examination (general)(routine)	Routine postpartum follow-up	Stroke (infarct)	Chronic renal insufficiency, stage iii (moderate)
Impaired glucose tolerance	Peripheral venous insufficiency	Medical care complication	Allergy, unspecified	Headache	Schizophrenia, unspecified	TIA	CKD stage 2 (EGFR 60–89)
Personal history of long-term (current) use of other medicaments, insulin	Arthralgia	De quervain’s tenosynovitis	Anaemia, unspecified	Heart disease, unspecified	Severe depressive episode without psychotic symptoms, not specified as arising in the postnatal period	Hypertension (diet only)	Osteopenia
Type 1 diabetes mellitus without complication	Venous embolism and thrombosis	Frozen shoulder	Arthropathy	Hereditary and idiopathic neuropathy, unspecified	Special screening	Epilepsy	Achilles tendinitis
Type 2 diabetes mellitus	Vomiting as reason for care in pregnancy	Disorder of gallbladder	Myalgia, site unspecified	Hyperlipidaemia	Special screening examination, unspecified	Sprain/strain	Cerebral palsy
Type 2 diabetes mellitus without complication	Complication related to pregnancy	Congenital anomaly of musculoskeletal system	Arthrosis, unspecified, site unspecified	Hyperlipidaemia, unspecified	Sprain, strain	Well women clinic	Arrhythmias
Unspecified diabetes mellitus with background retinopathy	Unwanted pregnancy	Back pain	Atherosclerosis of arteries of extremities	Hyperplasia of prostate	Stroke	Hyperlipidemia (diet only)	Thyroiditis
Unspecified diabetes mellitus with hypoglycaemia	Cobalamin deficiency	Dyslipidaemia	Atrial fibrillation	Hypertension	Stroke, not specified as haemorrhage or infarction	Non–DM nephropathy–incipient	Gall bladder disease
Impaired fasting glucose(IFG)	Folic acid deficiency	Itch	Atrial fibrillation and flutter	Hypothyroidism, unspecified	Supervision of normal pregnancy, unspecified	Other screening/growth monitoring. Questionnaires	Adverse effect, medication, chemical
DM retinopathy	Lateral Epicondylitis (Tennis Elbow)	Low Back Pain	Back Ache	IHD (ischaemic heart disease)	Tendency To Fall, Nec	Insomnia	Iron Deficiency
Impaired Glucose Tolerance(IGT)	Synovitis and tenosynovitis	TIA (transient ischaemic attack)	Bell’s palsy	Ill-defined condition	Thalassaemia, unspecified	Med exam/investigations	Menopausal disorders
Dm neuropathy	Tendinitis	Mammogram abnormal	Benign neoplasm of unspecified site	Inappropriate diet and eating habits	Thyrotoxicosis, unspecified	Schizophrenia	Family planning
DM nephropathy - ESRF on dialysis	Transient ischemic attack	Disease of circulatory system	Breast lump	Isolated proteinuria	Tobacco use, current	Osteoporosis	Pes planus
DM type i on medication	Fatty liver	Osa (obstructive sleep apnoea)	Cardiac arrhythmia, unspecified	Liver disease, unspecified	Transient cerebral ischaemic attack, unspecified	Antenatal care	Down’s syndrome
DM type ii on medication	Neck ache	Palpitations	Carpal tunnel syndrome	Loss of consciousness of unspecified duration	Trigeminal neuralgia	Acute ischemic heart disease	Other renal disorders
DM nephropathy - overt	Benign neoplasm	Metastatic malignant neoplasm	Other cvs conditions	Malignant neoplasm	Unspecified adverse effect of drug or medicament	Health education	Unspecified mental retardation without mention of impairment of behaviour
DM nephropathy - incipient	Well adult exam	Thyroid nodule	Chest pain, unspecified	Malignant neoplasm without specification of site	Unspecified dementia	Malignant neoplasms	Adjustment disorders
DM type ii (diet only)	Fracture neck of femur	Depressive illness	Chronic ischaemic heart disease, unspecified	Menopausal and perimenopausal disorder, unspecified	Unspecified disorder of bone density and structure, site unspecified	Anemia (except thal.)	Bipolar affective disorder, unspecified
Current use of insulin	Abnormal bone density screening	Stroke, haemorrhagic	Chronic nephritic syndrome, unspecified	Menopausal and postmenopausal disorder	Unspecified dorsalgia, site unspecified	Code not in dimension	Unspecified complication of procedure
Diabetes mellitus with incipient diabetic nephropathy	Idiopathic peripheral neuropathy	Prenatal consult	Chronic liver disease	Mental and behavioural disorders due to use of alcohol, acute intoxication	Unspecified lump in breast	Travel clinic	Contact with and exposure to other communicable diseases
Diabetes mellitus with retinopathy	Benign neoplastic disease	Chronic glomerulonephritis	Condition originating in the perinatal period, unspecified	Migraine, unspecified	Unspecified mental disorder due to brain damage and dysfunction and to physical disease	Stroke (haemorrhage)	Need for immunisation against unspecified combinations of infectious diseases
Impaired fasting glucose	Parkinson disease	Sprain and strain	Congenital malformation of heart, unspecified	Mild cognitive disorder	Unspecified nonorganic psychosis	Dementia	Radial styloid tenosynovitis [de quervain]
Hypoglycaemia	Dietary counselling and surveillance	Optional surgery	Congenital malformation of musculoskeletal system, unspecified	Neurotic disorder, unspecified	Unspecified osteoporosis, site unspecified	Depression (others)	Other and unspecified abnormalities of gait and mobility
Type 2 diabetes mellitus with hyperosmolarity with coma	Non-compliance with treatment	Encounter for postnatal visit	Congestive heart failure	Nutritional deficiency, unspecified	Unspecified synovitis and tenosynovitis, site unspecified	Non – dm nephropathy – overt	Unspecified harmful use of non-dependence producing substance
Diabetes mellitus, type ii	Cognitive dysfunction	Female infertility	Arthralgia & myalgia	Obesity	Chronic ischemic heart disease	Parkinsonism	Examination for adolescent development state
Diabetes mellitus	Trigger finger	Complication of the puerperium, postpartum	Contusion of unspecified body region	Obesity due to excess calories	Thyrotoxicosis	Migraine	Other problems related to housing and economic circumstances
Diabetic kidney disease	Mood disorder	Engorgement of breasts associated with childbirth, delivered	Counselling, unspecified	Obesity, unspecified	Backache	Preventive measures/immunisation child	Other specified postprocedural states;previously initiated endodontic therapy completed
IFG (impaired fasting glucose)	Hyperthyroidism	Subfertility of couple	Delayed milestone	Osteoarthritis	Anxiety	Headache, not specified	Other specified prophylactic measures
Type 1 diabetes mellitus	Thalassaemia	Mood and affect disturbance	Depressive episode, unspecified, not specified as arising in the postnatal period	Other amnesia	Benign neoplasms	Drp	Persistent delusional disorder, unspecified
Type 2 diabetes mellitus with hyperosmolar coma	Allergic drug reaction	Injured in road traffic accident	Disease of blood and blood-forming organs, unspecified	Other and unspecified disorders of breast associated with childbirth, without mention of attachment difficulty	Head injury	Hernia	Pregnancy-related condition, unspecified
Hypoglycaemia associated with diabetes	Elective surgical procedure	Gad (generalised anxiety disorder)	Disease of gallbladder, unspecified	Other and unspecified disorders of circulatory system	Ccf	Follow-up exam.	Cerebral palsy, unspecified
Diabetic retinopathy	Disorder of brain	Normal psychiatric assessment	Dislocation, sprain and strain of unspecified body region	Other examinations for administrative purposes	Spinal disorder	Nephritis (eg glomerulonephritis)	Cognitive impairment
DM (diabetes mellitus)	Major depression	Well child check	Disorder of brain, unspecified	Other general symptoms and signs	Psychosis	Other cns conditions	Postoperative follow-up
Diabetic neuropathy	Memory impairment	Encounter for examination for adolescent development state	Disorder of heart	Other specified counselling	Hyperlipidemia on medication	Rheumatoid arthritis	Myalgia
Long term current use of insulin	Stage 4 chronic kidney disease	Orthostatic hypotension	Disorders of initiating and maintaining sleep [insomnias]	Other specified disorders of breast	Stroke (not specified)	Nephritis, nephropathy, unspecified	Delayed developmental milestones
T2DM (type 2 diabetes mellitus)	Persistent delusional disorder	Asymptomatic human immunodeficiency virus [HIV] infection status	Dizziness and giddiness	Overweight	Thalassemia	Hypertension on medication	General counselling and advice for contraceptive management
IGT (impaired glucose tolerance)	Smoker	Venous insufficiency (chronic) (peripheral)	Down’s syndrome, unspecified	Parkinson’s disease	Other deformities of ankle and foot	BPH	Closed fracture
Type 2 diabetes mellitus with complications	Ischemic cerebrovascular accident (cva)	Screening for condition	Elevated blood pressure reading without diagnosis of hypertension	Peripheral vascular disease	Pvd	Congenital heart anomaly	Vertigo
Complication of procedure	Concussion	Disorder of endocrine system	Elevated blood-pressure reading, without diagnosis of hypertension	Peripheral vascular disease, unspecified	Nutritional def.	Depression (major)	Fall
Bipolar disorder	Deep vein thrombosis	Nutritional deficiency disorder	Embolism and thrombosis of unspecified vein	Personal history of noncompliance with medical treatment and regimen	Other blood disorders	Complication of medical care	Adverse effect of drug or medicament
Heart failure	Anxiety state	Erroneous encounter--disregard	Encounter for follow-up in outpatient clinic	Personal history of other mental and behavioural disorders	Glomerulonephritis	Basic health screen	ESRF (end stage renal failure)
Inflammatory arthropathy	Strain of knee	Anaemia	Endocrine disorder, unspecified	Plantar fascial fibromatosis	Other msk conditions	Disorder of synovium, tendon & bursa	carrier of viral hepatitis b
Cerebrovascular accident (CVA)	Dysfunction, psychosexual	Chronic ischaemic heart disease	Epilepsy, unspecified, without mention of intractable epilepsy	Plantar fasciitis	Giddiness, not specified	Other endocrine diseases	unspecified viral hepatitis without hepatic coma
End stage chronic kidney disease	Peripheral neuropathy	Allergy	Essential (primary) hypertension	Polyarthrosis, unspecified	Fractures	Drug/alcohol abuse	Hep B carrier follow-up
Congenital abnormality	Lipid disorder	Disorder of breast	Fatty (change of) liver, not elsewhere classified	Polyneuropathy, unspecified	Follow-up post surg	Need for immunisation against influenza	Hepatitis B carrier
Psoriatic arthropathy	Follow up	Disorder of cellular component of blood	Female infertility, unspecified	Problems related to unwanted pregnancy	Other psychiatric conditions	Encounter for gynecological examination	Hepatitis B infection
Disorder of thyroid	Limb ischaemia	Chest pain	Follow-up examination after unspecified treatment for other conditions	Procedure for purposes other than remedying health state, unspecified	Bunion/hallux valgus	Chronic kidney disease, unspecified	Disappearance and death of family member
CKD (chronic kidney disease)	Nonrheumatic aortic valve stenosis	Gout	Fracture of unspecified body region, closed	Prophylactic measure, unspecified	Hypothyroidism	ckd stage 3 or 4 (EGFR 15–59)	lack of physical exercise
Lymphadenopathy	Alcohol abuse	Gout, unspecified, site unspecified	General counselling and advice on contraception	Proteinuria	Graves’ disease	CKD stage 5/ESRF (EGFR < 15)	complication of surgical and medical care, unspecified
Food poisoning	Hypokalaemia	Acquired absence of foot and ankle	General medical examination	Rheumatoid arthritis, unspecified, site unspecified	Chest pain nos	Renal failure, chronic	Thalamic haemorrhage
Postural hypotension	Acquired absence of leg above knee	Generalised anxiety disorder	Risk for falls	Bipolar disorders	CKD stage 4 (egfr 15–29)		

**Table A2:** Multivariate logistic regression for factors predisposing to antibiotic prescriptions for undefined conditions, 2018–2021.

	B	SE	*p*-Value	OR	95% CI	
					Lower	Upper
Gender (Female)	0.111	0.016	<0.001	1.12	1.08	1.15
Race *			<0.001			
Indian	−0.044	0.027	0.104	0.957	0.908	1.01
Malay	−0.085	0.024	<0.001	0.919	0.877	0.963
Others	0.101	0.032	0.002	1.11	1.04	1.18
Age	0.005	0.001	<0.001	1.005	1.004	1.006
Diabetes mellitus	0.295	0.020	<0.001	1.34	1.29	1.40
Chronic kidney disease	0.272	0.022	<0.001	1.31	1.26	1.37
Physician Training (Locally trained)	−0.001	0.018	0.972	0.999	0.965	1.04
Years of physician experience	−0.007	0.001	<0.001	0.993	0.991	0.995
Family physician	0.150	0.018	<0.001	1.16	1.12	1.20
Place of practice ^+^			<0.001			
Clinic A	0.166	0.027	<0.001	1.18	1.12	1.25
Clinic B	0.087	0.027	0.001	1.09	1.04	1.15
Clinic C	−0.148	0.029	<0.001	0.862	0.815	0.912
Clinic D	0.028	0.029	0.333	1.03	0.972	1.09
Constant	−2.46	0.037	<0.001	0.085		

* compared to Chinese. ^+^ compared to Clinic E. B—logistic regression coefficient. SE—standard error. OR—odds ratios. 95% CI—95% confidence intervals.

**Table A3:** Multivariate logistic regression for factors contributing to Watch group antibiotic prescriptions, 2020–2021.

	B	SE	*p*-Value	OR	95% CI	
					Lower	Upper
Gender (Male)	0.233	0.032	<0.001	1.26	1.19	1.35
Race *			0.243			
Indian	−0.046	0.051	0.359	0.955	0.864	1.05
Malay	−0.064	0.046	0.159	0.938	0.858	1.03
Others	0.059	0.059	0.318	1.06	0.945	1.19
Age	0.007	0.001	<0.001	1.007	1.005	1.009
Diabetes mellitus	−0.067	0.041	0.101	0.935	0.863	1.01
Chronic kidney disease	0.018	0.043	0.672	1.02	0.936	1.11
Training (Locally trained)	0.197	0.032	<0.001	1.22	1.14	1.30
Years of physician experience	0.008	0.002	<0.001	1.008	1.005	1.011
Family physician	0.155	0.034	<0.001	1.17	1.09	1.25
Place of practice ^+^			<0.001			
Clinic A	0.104	0.049	0.033	1.11	1.01	1.22
Clinic B	−0.279	0.051	<0.001	0.757	0.684	0.837
Clinic C	0.101	0.048	0.037	1.11	1.01	1.22
Clinic D	−0.379	0.053	<0.001	0.684	0.616	0.760
Visit diagnoses ^^^			<0.001			
Dental	−0.116	0.194	0.551	0.891	0.609	1.30
ENT	1.36	0.088	<0.001	3.91	3.20	4.64
Eye	0.225	0.195	0.249	1.25	0.854	1.83
Gastrointestinal	3.35	0.086	<0.001	28.5	24.1	33.8
Genitourinary	2.32	0.058	<0.001	10.2	9.07	11.4
Infectious diseases	1.06	0.394	0.007	2.88	1.33	6.23
Multiple diagnoses	1.82	0.103	<0.001	6.18	5.05	7.56
Respiratory	2.47	0.058	<0.001	11.9	10.6	13.3
Undefined	1.59	0.064	<0.001	4.88	4.31	5.52
Constant	−4.58	0.086	<0.001	0.010		

* compared to Chinese. ^+^ compared to Clinic E. ^^^ compared to skin conditions. B—logistic regression coefficient. SE—standard error. OR—odds ratios. 95% CI—95% confidence intervals.

**Table A4:** Topical antibiotic prescriptions with skin and non-skin related diagnoses, 2018–2021.

Topical Antibiotics	2018, *n* = 14,558	2019, *n* = 14,445	2020, *n* = 14,359	2021, *n* = 14,809
Skin related diagnoses	9436 (64.8%)	9349 (64.7%)	9265 (64.5%)	8875 (59.9%)
Non-skin related diagnoses	5122 (35.2%)	5096 (35.3%)	5094 (35.5%)	5934 (40.1%)

**Table A5:** Multivariate logistic regression for factors contributing to topical skin antibiotics with irrelevant diagnoses, 2018–2021.

	B	SE	*p*-Value	OR	95% CI	
					Lower	Upper
Gender (Female)	0.172	0.018	<0.001	1.19	1.15	1.23
Race *			<0.001			
Indian	−0.171	0.031	<0.001	0.843	0.793	0.897
Malay	−0.369	0.030	<0.001	0.691	0.652	0.733
Others	−0.229	0.042	<0.001	0.795	0.732	0.864
Age	0.013	0.001	<0.001	1.013	1.012	1.015
Diabetes mellitus	0.405	0.021	<0.001	1.50	1.44	1.56
Chronic kidney disease	0.211	0.023	<0.001	1.23	1.18	1.29
Training (Locally trained)	0.066	0.019	<0.001	1.07	1.03	1.11
Years of physician experience	−0.002	0.001	0.101	0.998	0.996	1.000
Family physician	0.094	0.020	<0.001	1.10	1.06	1.14
Place of practice ^+^			<0.001			
Clinic A	0.343	0.033	<0.001	1.41	1.32	1.50
Clinic B	0.296	0.032	<0.001	1.35	1.26	1.43
Clinic C	−0.041	0.033	0.213	0.960	0.899	1.02
Clinic D	0.227	0.033	<0.001	1.26	1.18	1.34
Constant	−1.79	0.043	<0.001	0.166		

* compared to Chinese. ^+^ compared to Clinic E. B—logistic regression coefficient. SE—standard error. OR—odds ratios. 95% CI—95% confidence intervals.

**Table A6:** Multivariate logistic regression for factors affecting dual topical and oral antibiotic prescriptions for skin conditions, 2018–2021.

	B	SE	*p*-Value	OR	95% CI	
					Lower	Upper
Gender (Female)	0.121	0.021	<0.001	1.13	1.08	1.18
Race *			<0.001			
Indian	−0.082	0.034	0.017	0.921	0.861	0.985
Malay	−0.128	0.030	<0.001	0.880	0.829	0.934
Others	−0.117	0.043	0.007	0.890	0.818	0.968
Age	−0.006	0.001	<0.001	0.994	0.993	0.995
Diabetes mellitus	0.053	0.027	0.046	1.06	1.001	1.11
Chronic kidney disease	−0.103	0.029	<0.001	0.902	0.852	0.954
Training (Locally trained)	−0.167	0.023	<0.001	0.846	0.809	0.885
Years of physician experience	0.017	0.001	<0.001	1.017	1.015	1.019
Family physician	0.148	0.023	<0.001	1.16	1.11	1.21
Place of practice ^+^			<0.001			
Clinic A	0.003	0.036	0.931	1.00	0.934	1.08
Clinic B	−0.021	0.034	0.531	0.979	0.916	1.05
Clinic C	0.444	0.035	<0.001	1.56	1.45	1.67
Clinic D	0.077	0.036	0.032	1.08	1.01	1.16
Constant	−0.604	0.046	<0.001	0.547		

* compared to Chinese. ^+^ compared to Clinic E. B—logistic regression coefficient. SE—standard error. OR—odds ratios. 95% CI—95% confidence intervals.
